# Differential COVID-19 Symptoms Given Pandemic Locations, Time, and Comorbidities During the Early Pandemic

**DOI:** 10.3389/fmed.2022.770031

**Published:** 2022-01-28

**Authors:** Yang Wang, Fengwei Zhang, J. Brian Byrd, Hong Yu, Xianwei Ye, Yongqun He

**Affiliations:** ^1^Guizhou University School of Medicine, Guiyang, China; ^2^NHC Key Laboratory of Immunological Diseases, Department of Pulmonary and Critical Care Medicine, Guizhou Provincial People's Hospital and People's Hospital of Guizhou University, Guiyang, China; ^3^Division of Cardiovascular Medicine, Department of Internal Medicine, University of Michigan Medical School, Ann Arbor, MI, United States; ^4^Unit for Laboratory Animal Medicine, Department of Microbiology and Immunology, Center for Computational Medicine and Bioinformatics, University of Michigan Medical School, Ann Arbor, MI, United States

**Keywords:** COVID-19, symptom, phenotype, comorbidity, early pandemic, ontology, Human Phenotype Ontology (HPO), Coronavirus Infectious Disease Ontology (CIDO)

## Abstract

**Background:**

COVID-19 pandemic is disaster to public health worldwide. Better perspective on COVID's features early in its course–prior to the development of vaccines and widespread variants–may prove useful in the understanding of future pandemics. Ontology provides a standardized integrative method for knowledge modeling and computer-assisted reasoning. In this study, we systematically extracted and analyzed clinical phenotypes and comorbidities in COVID-19 patients found at different countries and regions during the early pandemic using an ontology-based bioinformatics approach, with the aim to identify new insights and hidden patterns of the COVID-19 symptoms.

**Results:**

A total of 48 research articles reporting analysis of first-hand clinical data from over 40,000 COVID-19 patients were surveyed. The patients studied therein were diagnosed with COVID-19 before May 2020. A total of 18 commonly-occurring phenotypes in these COVID-19 patients were first identified and then classified into different hierarchical groups based on the Human Phenotype Ontology (HPO). This meta-analytic approach revealed that fever, cough, and the loss of smell and taste were ranked as the most commonly-occurring phenotype in China, the US, and Italy, respectively. We also found that the patients from Europe and the US appeared to have more frequent occurrence of many nervous and abdominal symptom phenotypes (e.g., loss of smell, loss of taste, and diarrhea) than patients from China during the early pandemic. A total of 22 comorbidities, such as diabetes and kidney failure, were found to commonly exist in COVID-19 patients and positively correlated with the severity of the disease. The knowledge learned from the study was further modeled and represented in the Coronavirus Infectious Disease Ontology (CIDO), supporting semantic queries and analysis. Furthermore, also considering the symptoms caused by new viral variants at the later stages, a spiral model hypothesis was proposed to address the changes of specific symptoms during different stages of the pandemic.

**Conclusions:**

Differential patterns of symptoms in COVID-19 patients were found given different locations, time, and comorbidity types during the early pandemic. The ontology-based informatics provides a unique approach to systematically model, represent, and analyze COVID-19 symptoms, comorbidities, and the factors that influence the disease outcomes.

## Introduction

Since December 2019, the COVID-19 pandemic has caused an unpredictable and catastrophic disaster worldwide. COVID-19 is caused by SARS-CoV-2, a human coronavirus that was first found in Wuhan, China, and now worldwide. Other than the Wuhan strain, different variants of the SARS-CoV-2 virus, such as Alpha and Delta variants, have been identified. As of December 27, 2021, the pandemic has caused ~280 million confirmed cases and more than 5.4 million deaths globally. To better understand and control COVID-19, it is critical to study the host-coronavirus interactions, including the various symptoms or phenotypes occurring in COVID-19 patients.

While the common symptoms of COVID-19 patients are generally similar between studies, differences have also been found (1–4). The common symptoms include fever, cough, myalgia and fatigue; some patients also develop digestive symptoms such as nausea, vomiting, abdominal pain and diarrhea. Some patients have suffered the new loss of smell or taste as a symptom of COVID-19. However, these symptoms appeared to have varied over time, viral variant, patient condition, and geographically. COVID-19 patients with comorbidities tend to have poorer outcomes and higher mortality. Comorbidities here refers to the simultaneous presence of some disease(s) or condition(s) in a patient when COVID-19 occurs in the patient. Hypertension, diabetes, and cardiovascular diseases are a few examples of the most common comorbidities commonly reported in patients infected with COVID-19 (5). To better understand and control the pandemic, it is important to systematically investigate various types of COVID-19 disease symptoms and comorbidities under different conditions and at different stages. The ignorance of the patterns hidden in these phenomena would pose us some huge risks without prompt actions.

Given the extensive reports and complexity of the COVID-19 symptoms in patients with different backgrounds, it is crucial to standardize the results and conditions of these studies in order to achieve consistent and integrative results. Ontology has emerged to play a significant role in standard data representation, classification, integration, and analysis. Basically, an ontology is a human- and computer-interpretable vocabulary of terms and relations that represents entities and how they interact in a specific domain. The Human Phenotype Ontology (HPO) is a standardized vocabulary for phenotypic abnormalities and clinical features encountered in human disease. Each term in the HPO describes a phenotypic abnormality, such as fever, cough, pneumonia, etc. HPO currently contains over 13,000 terms (6). The Coronavirus Infectious Disease Ontology (CIDO) is a newly developed community-based ontology in the domain of coronavirus infectious diseases, including SARS, MERS, and COVID-19 (7). CIDO systematically represents and interlink these diseases with phenotypes, disease etiology, transmission, drugs, vaccines, etc. CIDO has been proven to be effective in modeling and representation of anti-coronavirus drugs (8). CIDO reuses HPO terms for classifying the phenotypes and symptoms associated with coronavirus diseases including COVID-19.

In this study, we focused on systematic annotation, integration, and analysis of various COVID-19 symptoms that were identified in the early stage of the COVID-19 pandemic. Specifically, we systematically collected, annotated, and analyzed COVID-19 clinical phenotypes and comorbidities that occurred in patients infected with COVID-19 during the early stage of the pandemic. Our study used the data primarily extracted from journal and preprint articles published by August 2020. The data reported in these publications were collected by early May 2020.

Our study considers May 2020 as an important time cutoff of the early pandemic development. Historically, it had been approximately half a year from the first case report in December 2019–May 2020. On May 2, the WHO renewed its previous emergency declaration calling the pandemic a global health crisis. The COVID-19 death toll in the U.S. surpassed 100,000 in May 2020, a critical milestone in the pandemic history. Meanwhile, while many variants of SARS-CoV-2 appeared to exist by early May 2020 (9), the major variants of concern (e.g., Alpha, Beta, Delta strains) of SARS-CoV-2 defined by the WHO (10) were not yet reported. For example, the virus variant lineage B.1.1.7 was first detected in October 2020 in UK, and it was later labeled as the Alpha strain and became the first variant under investigation in December 2020 by the WHO. The Alpha variant was reported to have 40–90% increased transmissibility (11) and lethality (12). The Beta variant was first identified at South Africa in October 2020 and likely first emerged in July or August 2020 (13). No COVID-19 vaccine was licensed or provided at the early stage. By selecting the early stage of pandemic, we were able to focus on the other variables on the symptoms than major highly transmissible viral variants and vaccination. Targeting our study on the early stage, we could also focus on the relatively less but more manageable number of publications, with the aim to find distinct patterns at the early stage, permitting useful and relevant discovery.

In addition, to systematically understand the changing trend of the occurrence and development of patients' symptoms, we applied the HPO and CIDO ontologies to classify these phenotypes, comorbidities, and the relations between each of them and the disease. Our study identified many shared and differential phenotype patterns in COVID-19 patients from countries in different regions of the world. Since all the symptoms are included in HPO and the term “symptom” is more naturally and medically used, in this paper we have prioritized the usage of the term “symptom” later in this paper.

## Methods

### Related Article Search and Collection

Peer-reviewed journal articles from December 2019 to August 2020 in PubMed and preprint bioRxiv and medRxiv articles were searched to identify relevant articles. The keywords used to identify the COVID-19 disease included “COVID-19,” “2019-nCoV,” and “SARS-CoV-2.” The keywords used to identify symptoms included “Clinical,” “Phenotype,” “Symptom,” “Character,” and specific symptoms such as “loss of smell” and “loss of taste.” The countries searched in our study included “China,” “the United States,” “Iran,” and many European Countries including “United Kingdom,” “Italy,” “France,” and “Germany,” etc.

Inclusion and exclusion criteria were preset in our study. Studies were included when the following inclusion criteria were met: (1) articles were original English-language reports; (2) COVID-19 patient cases were laboratory-confirmed; (3) the clinical data of COVID-19 patients were first-handed data; (4) detailed information about clinical phenotypes was provided; and (5) articles were published before August 2020. Our study showed that the clinical data covered in all our identified articles were collected by early May 2020, which represented the cutoff of the early stage of the pandemic. The reasons of this selection are described in the Introduction.

As an exclusion criterion, case reports of individual cases were excluded in this study. In one specific three-country (China, Italy, and USA) comparison, the infection cases in children and pregnant women were not included, and those articles with <50 patients each were excluded. However, for a more general scope study, these two exclusion criteria were not applied due to the complexity of the datasets covered in various articles.

Although the data from related bioRxiv and medRxiv papers were initially extracted and used, we later found that all the bioRxiv and medRxiv preprint papers used in our study had their corresponding journal articles, and sometimes the data in the formal journal articles were updated compared to their preprint versions. In this case, we updated our data records at a later stage to use the newly reported data in the formal journal articles. We also ensured that no double counting occurred in our study.

### Symptom Extraction, Mapping to HPO, and Hierarchical Classification

COVID-19-related symptoms and comorbidities were manually identified from the articles. The main source of the information came from the tables and Supplementary Materials provided in the articles. The extracted data were initially organized in a pre-defined Excel worksheet (See Supplementary Files 1–3). The extracted results were peer-reviewed by two members in the project.

The identified symptoms and comorbidities were mapped to the terms in the Human Phenotype Ontology (HPO) (14). The Ontobee ontology browser (http://www.ontobee.org) (15) was primarily used to search the phenotype and symptom terms from the HPO. Synonyms (such as fever and pyrexia) might be used in different articles. We ensured that these synonyms were mapped to the same HPO IDs (e.g., HPO_0001945).

To identify the hierarchical relations among the COVID-19 related symptoms and comorbidities detected in our literature mining, the Ontofox ontology term extraction tool (http://ontofox.hegroup.org) (16) was used. The settings of “includeComputedIntermediates” and “includeAllAxioms” were used in the Ontofox execution, leading to the extraction of additional high level HPO terms that are the ancestral terms of our identified specific symptoms and comorbidities. To display and analyze the extracted terms out of the Ontofox execution, the Protégé-OWL editor (17) was used.

### CIDO Ontology Modeling, Representation, and Analysis

The Coronavirus Infectious Disease Ontology (CIDO) was used as a platform for further COVID-19 symptom knowledge representation and analysis. Specifically, the Ontofox output described above, which included our identified HPO terms, was first imported to the CIDO to become a part of the CIDO. Further ontology modeling and representation were performed in the CIDO to add new relations that semantically link the phenotypes with other entities in CIDO.

The Protégé-OWL editor was used for ontology term display, classification, editing, and analysis.

CIDO is formatted using the Web Ontology Language (OWL; https://www.w3.org/OWL/) format, which is computer interpretable. Computational programs can be developed to automatically query the knowledge stored in the CIDO. For example, the CIDO knowledge represented in OWL can be queried using Description Logic (DL)-query or SPARQL Protocol and RDF Query Language (SPARQL). SPARQL scripts were developed to automatically query the CIDO knowledge using the Ontobee SPARQL program (http://www.ontobee.org/sparql) (15).

## Results

### Statistics of Surveyed and Analyzed Studies in the Literature

This study aimed to collect, annotate, and compare the clinical phenotype data across a range of studies of patients who were diagnosed with COVID-19 during the early stage of the pandemic, specifically, before August 2020. The early stage of the disease outbreak and pandemic is an important time period since there were no major viral variants spreading during the time, and vaccination had not started so that we do not need to consider the influence of vaccination on the phenotype changes. The focus on this initial stage would allow us to identify possible patterns of COVID-19 symptoms and detect possible demographic and environmental conditions that might have affected the disease patterns.

Our study initially identified 75 research articles that report COVID-19 symptoms. Using the inclusion and exclusion criteria as listed in the Methods section, a total of 48 research articles remained in our final list of study (Supplementary File 1). During our article survey and selection, we did not exclude articles based on any selection bias that might influence the results. In aggregate, these papers reported clinical results from 43,567 patients (Supplementary Files 1–3).

Our literature search found that clinical phenotypes of COVID-19 patients were first reported early in Asian countries such as China, South Korea, Japan, Singapore, and Iran, followed by European countries such as Italy, France, Germany, and the United States. We collected the data based on the chronological order and geographical characteristics of the emergence of the COVID-19 epidemic. For concise and focused reporting, we have chosen two strategies for presentation: one is to focus on representative countries, the other is to focus on specific regions.

### Classification of Common Phenotypes and Comorbidities in Early COVID-19 Patients

Using the methods detailed in the Methods section, a total of 18 common clinical symptoms were identified, including fever, cough, dyspnea, myalgia, arthralgia, chills, sore throat, headache, new loss of smell or taste, rhinorrhea, nasal congestion, diarrhea, abdominal pain, nausea and vomiting, fatigue, coagulation disorders (Supplementary Table 1) (2, 18–20). Lee et al. (21) reported laboratory evidence of coagulation disorders in ~20–55% of patients hospitalized for COVID-19. Although not strictly speaking a symptom, coagulation disorders were included in the phenotypic collection in view of their significant impact on patient outcomes. Note that some other symptoms, including hemoptysis, dizziness, tinnitus, etc., were not included in our collection this time because these symptoms were rarely reported in the literature and the number of cases reported was small.

**Figure 1:**
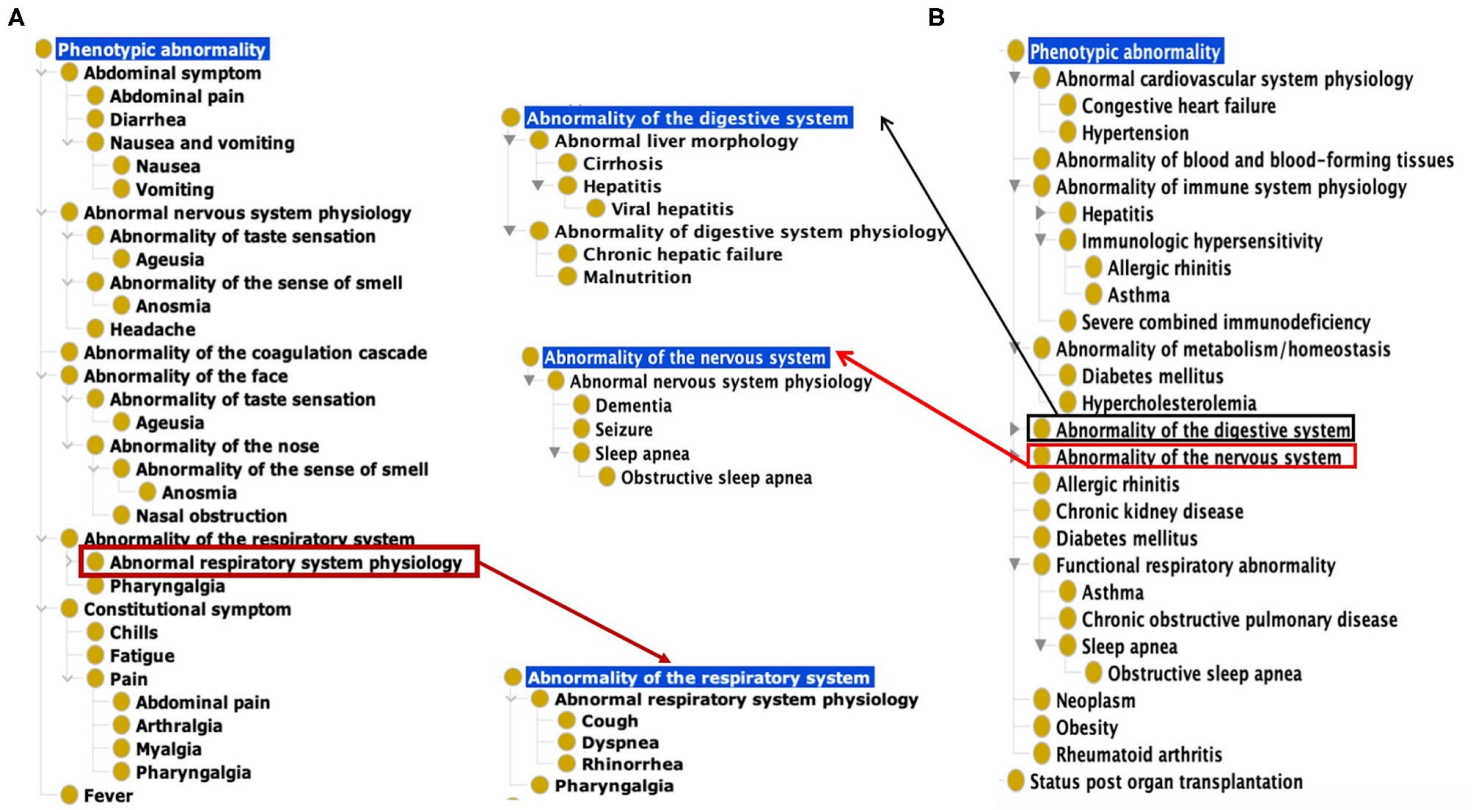
Common COVID-19 symptoms and comorbidities represented using the Human Phenotype Ontology (HPO). **(A)** 18 Common symptom phenotypes of COVID-19 based on HPO classification. The list of these 18 phenotypes is provided in Supplementary Table 1. **(B)** Hierarchical lay of 22 common comorbidity phenotypes based on HPO classification. The list of these 22 phenotypes is also provided in Supplementary Table 1.

Based on our ontological classification, these 18 common symptoms were classified to occur in different systems, including the respiratory system, gastrointestinal system, nervous system, constitutional system, and coagulation cascade (Figure 1). Pharyngalgia (i.e., sore throat) is an unpleasant sensation characterized by physical discomfort, which belongs to abnormal respiratory system physiology and constitutional symptom. Four types of pains, including abdominal pain, pharyngalgia, arthralgia, and myalgia, were frequently found in COVID-19 patients. These pains, together with chills and fatigue, belong to the constitutional symptoms. Fever, one common abnormal homeostasis symptom defined as elevated body temperature due to failed thermoregulation, is not classified with the above 4 systems (Figure 1).

Based on the categorization of pathophysiology, Ageusia (i.e., loss of taste) and Anosmia (i.e., loss of smell) belong to the abnormal nervous system physiology. Based on anatomic location, they belong to the category of abnormality of the face. The synonyms of ageusia include impaired taste sensation, absent sense of taste, and lost taste (Figure 1A). Anosmia's synonyms in HPO include lost smell and loss of smell. These symptoms newly arising within a patient are critical signs for diagnosing COVID-19 patients. The other abnormality of the face identified as a frequently occurring symptom was nasal obstruction. These categories defined by the pathophysiology and anatomic locations provide a hierarchical and more systematic view of the phenotypes of COVID-19. These classifications facilitate prompt disease location and identification of etiology.

Our literature survey identified 22 common comorbidities. Hypertension, diabetes, cardiovascular diseases, chronic pulmonary diseases are the most common comorbidities in COVID-19 patients. Other common comorbidities include obesity, chronic kidney, hepatitis B/C infection, chronic hepatic failure, cirrhosis, chronic neurological disease (e.g., seizures and dementia), and hematological system disease (e.g., abnormality of blood and blood-forming tissues) (22, 23). These comorbidities were classified to different hierarchical groups (Figure 1B). Specifically, these comorbidities were found to occur in various systems such as the cardiovascular system, blood, immune system, metabolism, digestive system, nervous system, kidney, respiratory system. Cirrhosis, viral hepatitis, and chronic hepatic failure belong to the digestive system. Dementia, seizure is the subclass of abnormal nervous system. Obesity and autoimmune deficiency comorbidities are also important markers for poor prognosis (Figure 1B).

### Differential Profiles of Symptoms in Early COVID-19 Patients

#### Differential Profiles of Symptoms in Early COVID-19 Patients in China, Italy, and the USA

**Figure 2:**
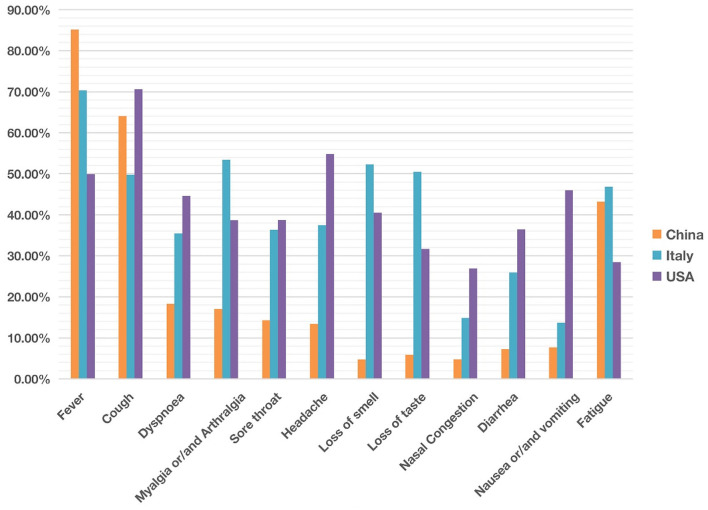
Comparison of common clinical symptoms of COVID-19 between China, Italy and the United States from December 2019 to May 2020. China was ranked the highest in fever, The USA was ranked the highest in cough, dyspnea, headache, diarrhea, and nausea/vomiting. Italy was ranked the highest in myalgia/arthralgia, loss of smell, loss of taste, and fatigue. Overall the COVID-19 symptoms in Italy and the USA appeared more similar than those in China. Each symptom has 0–100% of occurrence. See the data details in Table 1.

To examine how the COVID-19 symptoms varies depending on countries, we focused our study on the set of rich clinical data from China, Italy and the United States of America (USA). These three countries were first chosen because of the many reports on the clinical results from these three countries, and there appeared to be differences in clinical phenotypes in patients reported in these three countries as well. A total of 17 articles included 5 articles from China (1, 2, 24–26), 7 from Italy (27–33), and 5 from the US (34–38). As summarized in Table 1, these 17 papers annotated in our study covered 3,630 patients from different countries. The commonly reported 12 symptoms in these papers were listed in Table 1 and plotted in Figure 2 and Table 1.

**Table 1:** Comparison of common COVID-19 phenotypes in China, Italy and USA from December 2019 to May 2020.

	**China**	**Italy**	**USA**
Fever	1,406/1,651 (0.85)^*^	940/1,337 (0.7)	282/565 (0.50)
Cough	1,058/1,651 (0.64)	665/1,337 (0.5)	399/565 (0.71)
Dyspnea	252/1,377 (0.18)	236/665 (0.35)	252/565 (0.45)
Myalgia or/and Arthralgia	221/1,297 (0.17)	486/910 (0.53)	123/318 (0.39)
Sore throat	208/1,451 (0.14)	139/382 (0.36)	219/565 (0.39)
Headache	202/1,511 (0.13)	368/982 (0.37)	310/565 (0.55)
Loss of smell	13/274 (0.05)	699/1,337 (0.52)	229/565 (0.41)
Loss of taste	16/274 (0.06)	573/1,135 (0.5)	133/420 (0.32)
Nasal congestion	53/1,099 (0.05)	130/874 (0.15)	152/565(0.27)
Diarrhea	115/1,591 (0.07)	328/1,265 (0.26)	153/420 (0.36)
Nausea or/and vomiting	105/1,377 (0.08)	110/802 (0.14)	154/335 (0.46)
Fatigue	621/1,437 (0.43)	542/1,157 (0.47)	156/548 (0.28)

*The data were obtained by summarizing the results reported in 17 papers (see Supplementary Files 1, 2)*.

* *Represents 1,406 out of 1,651 (85%) COVID-19 patients who had the fever symptom*.

Our comparative study showed that the incidence of fever of COVID-19 patients was 85% in China, higher than in the other two countries (Figure 2). In contrast, 70 and 50% of the patients in Italy and the US had fever as a symptom. With an incidence of 43%, fatigue was also common in COVID-19 patients in China. Beyond fever, cough, and fatigue, the incidence of other symptoms (i.e., headache, diarrhea, nausea and vomiting etc.) in China was relatively lower, all below 20%. This finding accords with the results published in a paper (also included in our study) by Guan et al. (2), which summarized the clinical data from 1,099 COVID-19 patients from 552 hospitals in 30 provinces in China and found that the most common symptoms of COVID-19 in China were fever (88.7% during hospitalization) and cough (67.8%), whereas diarrhea was uncommon (3.8%).

COVID-19 patients in the US had 7 symptoms ranked most frequently reported out of the three countries: cough, dyspnea, sore throat, headache, nasal congestion, diarrhea, and nausea/vomiting. A total of 71% of patients in the US reported cough, and the numbers were 64 and 50% in China and Italy, respectively. The most dramatic difference appeared to be nausea/vomiting, with 46% in the US, 14% in Italy, and 8% in China. The incidence of headache also differed substantially among the three countries, with 55% in the US, 37% in Italy, and 13% in China.

COVID-19 patients in Italy showed four symptoms at a higher frequency compared with the other two countries: myalgia/arthralgia, loss of smell, loss of taste, and fatigue. A total of 52% of the patients in Italy reported loss of smell, and 51% reported loss of taste. In the US, 32 and 41% of patients reported loss of smell and taste, respectively. In contrast, in China, only 5% of the patients reported loss of smell, and 6% reported loss of taste (Table 1).

#### Differential Profiles of Nervous Symptoms in Early COVID-19 Patients From Different Countries and Regions

Instead of focusing on individual symptoms, we hypothesized that the analysis of symptoms as groups would identify new scientific insights. First, we analyzed the group of COVID-19-related nervous system symptoms, which includes loss of smell (anosmia), loss of taste (ageusia), and headache as shown in the HPO classification. Our combined analysis of all three symptoms, at the study/paper level instead of individual patient level, provided us with a unique approach to studying how the disease affects the nervous system.

**Figure 3:**
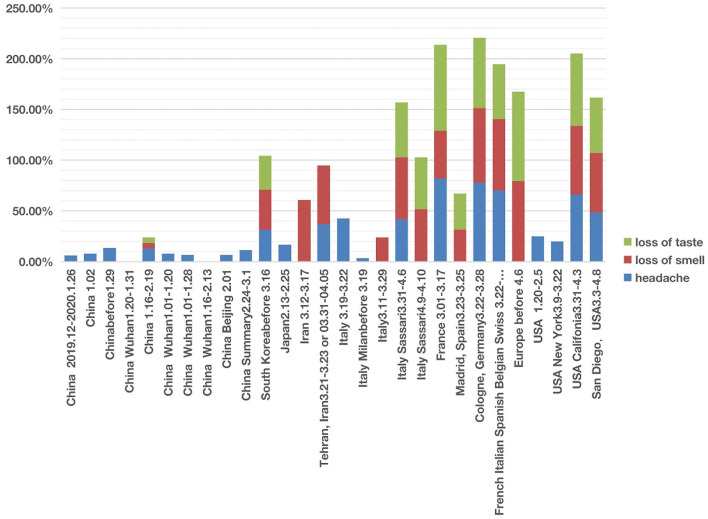
COVID-19 cases with three nervous system symptoms in different countries from December 2019 to May 2020. Three symptoms (i.e., headache, loss of smell, and loss of taste) were analyzed. Each symptom has 0–100% of occurrence.

In studies of patients in East Asian countries including China, South Korea, and Japan, all three nervous system symptoms were uncommon (Figure 3; Supplementary Files 1, 2). Among the three symptoms, headache was relatively common in these countries, with incidences ranging from 6 to 31.4%. However, except one article in China reporting hyposmia (i.e., reduced ability to smell) and hypogeusia (i.e., reduced ability to taste) (39), the other 9 Chinese articles did not report any cases of loss of smell and loss of taste. All these 10 Chinese articles covered patients with COVID-19 from December 2019 to February 2020. Another retrospective cohort study published in February 2021, which followed 1,172 COVID-19 patients discharged from Tongji Hospital in Wuhan between January and March 2020. The result was that 20.6% patients had loss of taste and 11.4% had loss of smell (40). The reduced or lost ability of smell or taste was not reported in a paper that collected the clinical data with the early six patients in Japan in 13–25 February 2020 (41) (Figure 3). However, an analysis of 213 patients in South Korea with mild COVID-19 during 12–16 March 2020 and assigned to specialized isolation facilities found that 39.5% of these patients reported hyposmia and 33.7% reported hypogeusia (42). It is still unclear whether the difference in smell and taste related symptoms was due to an issue of reporting instead of the reality. However, it appears that the report of smell and taste related symptoms became common after early March.

The nervous system symptom patterns in Iran, European countries (including Italy, France, Germany, and Spain) and USA appeared to overlap and differ in many ways from China according to the reports during the first stage of infection (Figure 3; Supplementary Files 1, 2). The major differences existed in the loss of smell and taste. The incidence of loss of smell in Iran ranged from 58 to 60.9% (3, 43). Two large European investigations (44, 45) identified the loss of smell as a key symptom in mild-to-moderate COVID-19 patients, and they noticed that the loss of smell was not associated with nasal obstruction and rhinorrhea. Females and young patients were more susceptible to having the symptoms of smell and taste losses. Five Italian articles reported nervous system symptoms (Figure 3). Two of these five studies only reported headache but not loss of smell or taste (1, 41). However, loss of smell (24–61%) was the major nervous system symptoms among the three other studies (26, 42, 43), and loss of taste (51–54%) was reported in two studies (30, 46). In addition, loss of taste and smell were widely reported in COVID-19 cases in France, Spain, Germany, Belgium, and Switzerland (47). A systematic study published in April 2020 concluded 80% loss of smell and 88% loss of taste in Europe (44).

The three nervous system symptoms (i.e., headache, loss of taste, and loss of smell) in four US studies were collected and analyzed (34, 48–50) (Figure 3). Two studies with hospitalized COVID-19 patients reported the incidence of headache ranging from 20 to 25%, but not loss of smell or taste (48, 49). Based on self-reported symptoms (34, 50), the other two studies reported high incidences of all the three symptoms, with headache ranging from 48 to 66%, loss of taste from 55 to 71%, and loss of smell from 59 to 68%. These two survey-based studies found that COVID-19 patients with general mild-to-moderate symptoms more likely had the loss of smell and taste symptoms.

#### Differential Profiles of Gastrointestinal Phenotypes in Early COVID-19 Patients From Different Countries and Regions

The primary abdominal symptoms included diarrhea, abdominal pain, nausea, and vomiting. With the help of the HPO classification (Figure 1), we analyzed these four abdominal symptoms together and compared the cases from different countries and regions.

**Figure 4:**
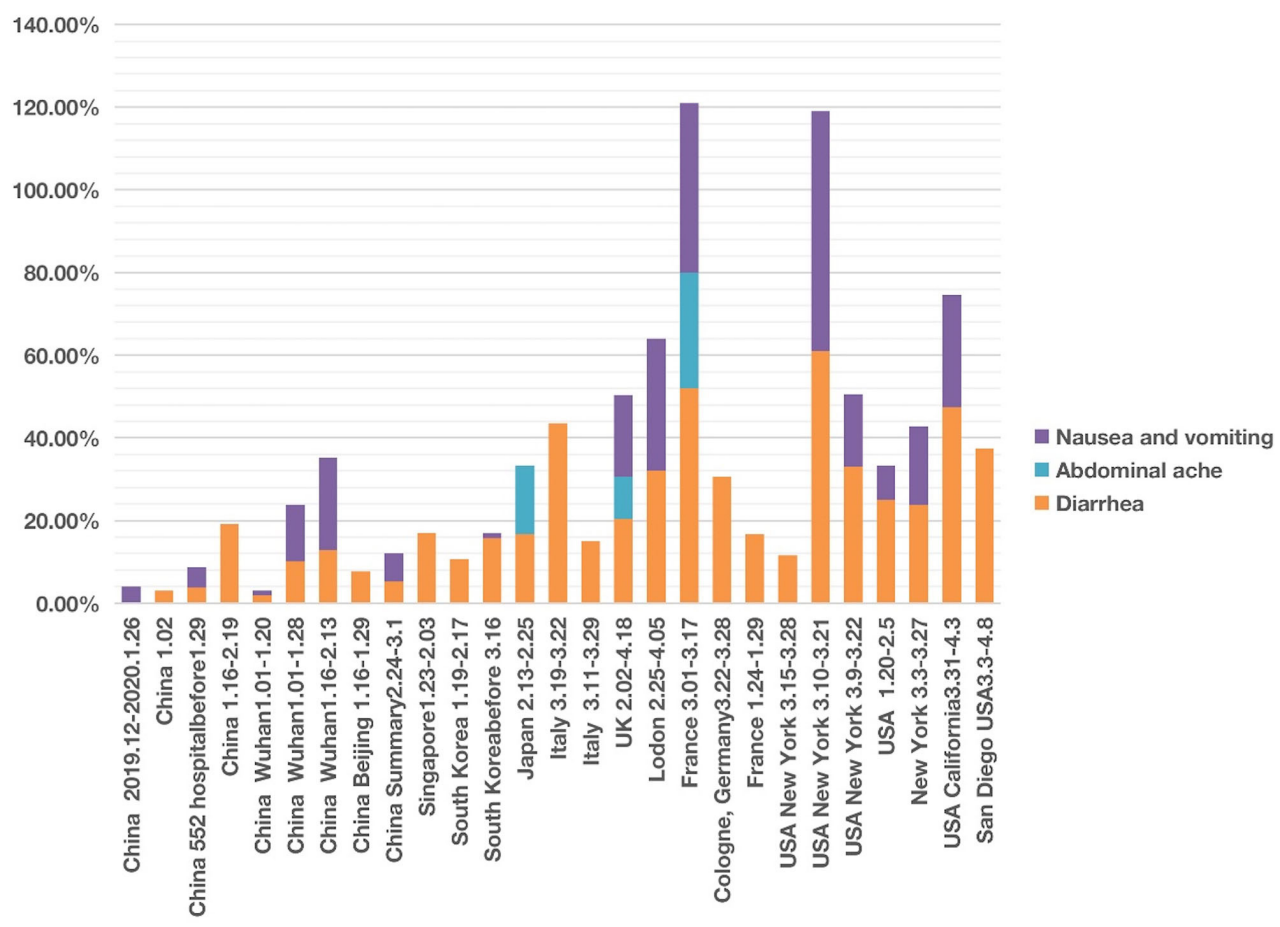
COVID-19 cases with four abdominal system symptoms in different countries from December 2019 to May 2020. Four symptoms (i.e., nausea, vomiting, abdominal pain, and diarrhea) were analyzed. Each symptom has 0–100% of occurrence.

We found that of the four digestive symptoms, diarrhea occurred in almost all countries, but the incidence in East Asian countries, including China, Singapore, South Korea, Japan, etc. was lower than 20%. In contrast, the incidence in Italy, the UK, France, Germany, USA generally ranged from 20 to 40%, and the highest proportion in one report of New York reached 61% (Figure 4; Supplementary Files 1, 2). The highest proportion of nausea and vomiting reported in China was 22.3% (24). However, the incidence in one report in New York reached 58% (51); the authors found that the patients with gastrointestinal symptoms (defined as diarrhea or nausea/vomiting) were significantly more likely to test positive than to test negative for COVID-19 (61 vs. 39%, *P* = 0.04). Among 393 COVID-19 patients in two New York hospitals (4), gastrointestinal symptoms seemed to be more common than in China.

After screening, we documented three articles that specifically reported abdominal pain of COVID-19 patients. One study in Japan reported an incidence of 16.67% (41). Another study conducted in France found the incidence of abdominal pain was 28.00% among 54 patients with anosmia (20). In the UK, the incidence of abdominal pain was 10.20% of 20,133 patients between 6 February and 19 April 2020 in 208 acute care hospitals (52).

The time appeared to be another factor related to the presence of abdominal symptom phenotypes. The different abdominal symptoms were infrequently reported in studies in January and February 2020. However, more digestive symptoms were shown in the middle-late of March and early April 2020 (Figure 4).

### Correlation Between Comorbidities and Severe Disease Outcomes

**Figure 5:**
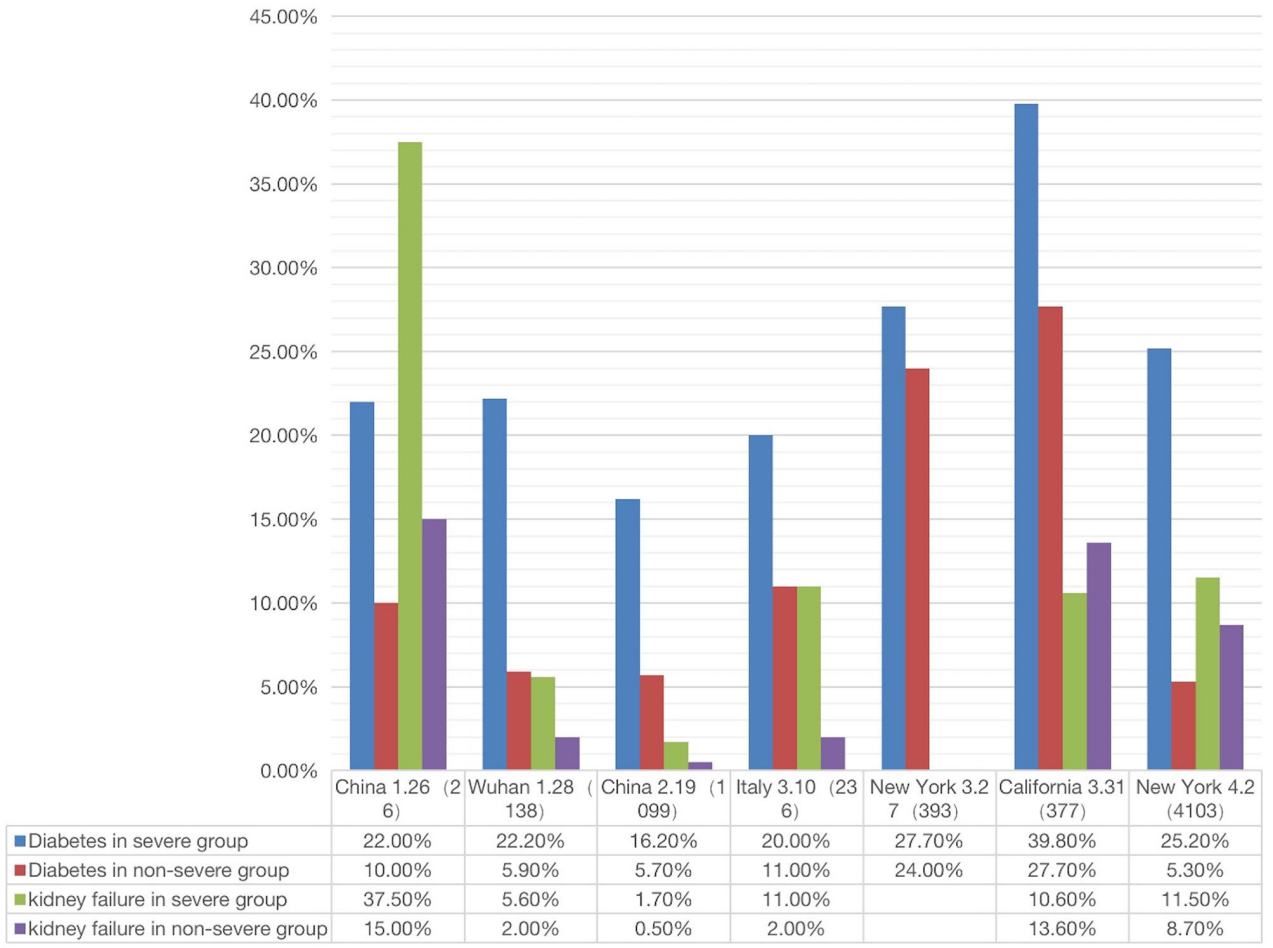
Correlation between two comorbidity phenotypes (diabetes and kidney failure) and two outcomes (severe or non-severe) (see Supplementary File 3). In severe disease patients, the incidence of diabetes or kidney failure was higher than that in non-severe patient groups (The X-axis is country/city, report date, number of cases).

To further study the relation between different comorbid phenotypes and disease outcomes, we reviewed the literature and compared the incidences of specific comorbid phenotypes in severe or non-severe COVID-19 patients. From the long list of comorbidities (Figure 1B), we chose diabetes and kidney disease for our further analysis (Figure 5). Zaki et al. showed that diabetes, hypertension, and cholesterol levels were significantly associated with COVID-19 severity, and kidney disease was strongly linked to the viral infection (53). Cheng et al. found that COVID-19 patients had a high prevalence of renal disease on admission and the development of acute kidney injury during hospitalization, and the kidney disease comorbidity was associated with increased in-hospital mortality (54). Therefore, diabetes and kidney disease appeared to be two independent risk factors for poor prognosis and intensive care unit admission in patients with COVID-19.

Given the poor prognosis of COVID-19 in patients with diabetes and kidney disease, we made a more systematic comparison of how the two diseases were related to the prognosis (i.e., severe or non-severe) of COVID-19 patients. The results from a total of 7 papers were summarized and analyzed (Figure 5). These 7 papers were selected since they met our inclusion criteria and included the incidences of COVID-19 comorbidities in both severe and non-severe patients (1, 2, 4, 19, 55–57).

As shown in Figure 5, the percentage of severe COVID-19 patients with diabetes was generally higher than that in non-severe patients. In all regions except California, the percentage of kidney disease of COVID-19 patients was also higher in severe patients than in non-severe patients. Specifically, the incidence of diabetes with severe or non-severe outcomes ranged from 16.20 to 39.8% or 5.7 to 27.7%, respectively (Figure 5). For each study, the fold change between severe and non-severe diabetes was usually over 2.0; even in California it was 1.15. The incidence of kidney disease of COVID-19 in the severe group ranged from 1.7 to 37.5%, in the non-severe group ranged from 2.0 to 15.0% (Figure 5). The average fold change between these patients was 2.7. In summary, the above results indicated that the patients with diabetes and kidney failure had worse outcomes compared with those without these comorbidities. It was reported that cytokine storm might be activated in diabetic patients, leading to poor prognosis and death (58). The exact mechanism deserves further investigation.

### CIDO Modeling, Query and Analysis of the COVID-19 Phenotypes and Comorbidities

**Figure 6:**
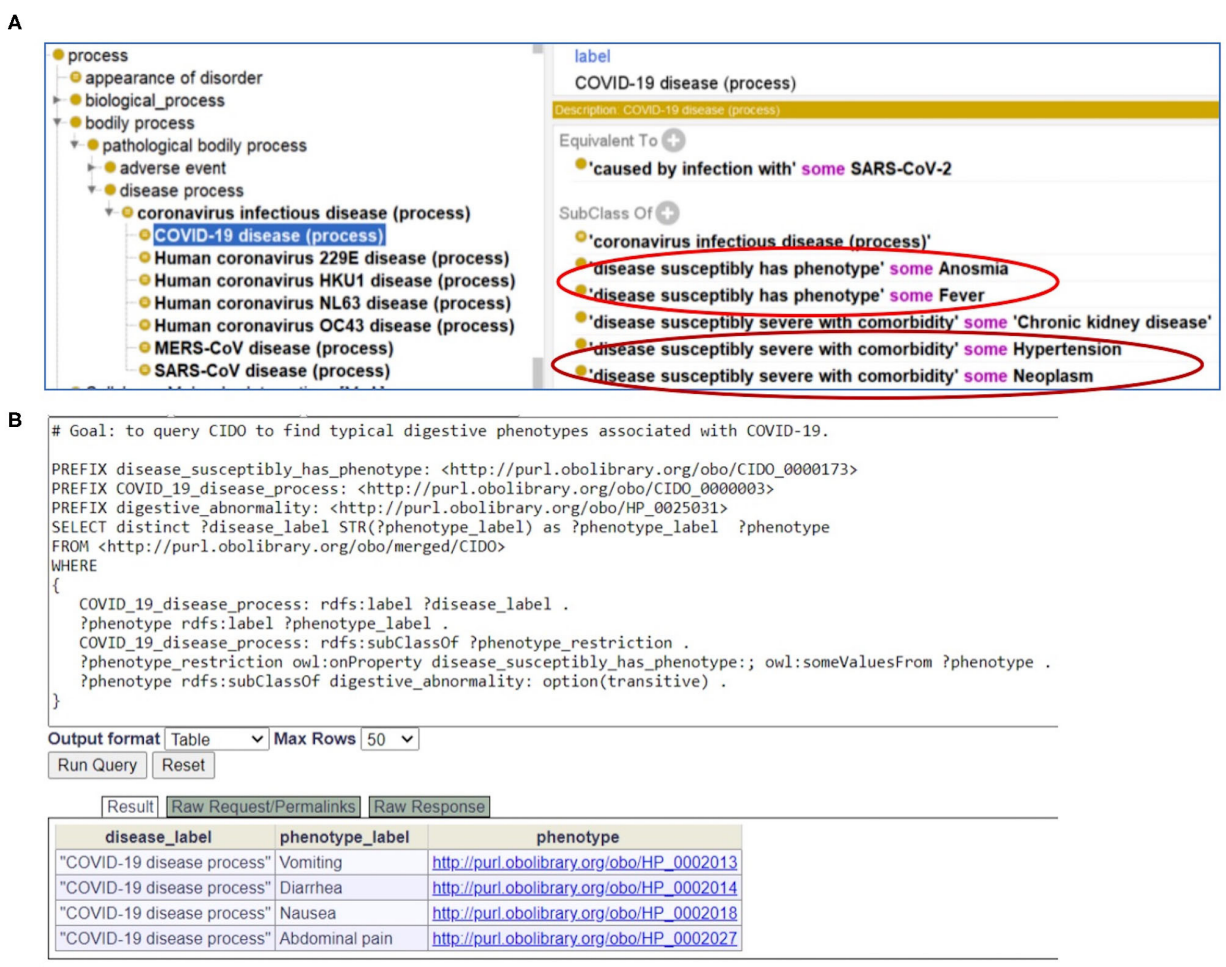
CIDO modeling and analysis of COVID-19 symptoms. **(A)** Generation and usage of two new relations in CIDO to link COVID-19 to symptoms or comorbidities. **(B)** SPARQL query of typical digestive phenotypes associated with COVID-19 disease (process). Four phenotypes (i.e., vomiting, diarrhea, nausea, and abdominal pain) were identified. The Ontobee SPARQL website (http://www.ontobee.org/sparql) was used to perform the query.

As a foundation of AI, ontology is computer-interpretable. To allow users, including bioinformaticians, to develop and use computational programs to automatically access our annotated data, we represented our annotated knowledge in the Coronavirus Infectious Disease Ontology (7). Different diseases have the potential or susceptibility to induce specific phenotypes in patients. To differentiate the relations between diseases and phenotypes, in CIDO we generated a new ontology relation called “disease susceptibly has phenotype.” With this relation, we can define the disease-phenotype relation such as the following (Figure 6A):

*COVID-19 disease (process): “disease susceptibly has phenotype” some Fever*.

This means that COVID-19 patients are prone to fever. Such representation semantically links the disease to various phenotypes.

Similarly, to link a coronavirus disease such as COVID-19 with a comorbidity with which the disease is likely to have a more severe outcome, we also generated a new relation named “disease susceptibly severe with comorbidity.” With this relation in CIDO, we can define the disease-comorbidity relation such as the following (Figure 6A):

*COVID-19 disease (process): “disease susceptibly severe with comorbidity” some hypertension*.

This relation indicates that COVID-19 patients with hypertension and other comorbidities are prone to poor disease outcomes and prognosis. Such axioms allow us to semantically separate those phenotypes that are new symptoms in COVID-19 patients and the other phenotypes that are comorbidities present in COVID-19 patients when they were infected with the SARS-CoV-2 virus.

With CIDO modeling and representation of the COVID-19 disease, symptoms of COVID-19, and comorbidities in COVID-19 patients, and the semantic relations among these terms, we are able to develop new methods to query and analyze the knowledge represented in the CIDO using ontology-based semantic tools. Figure 6B demonstrates how we can generate a SPARQL query to identify the four COVID-19-associated digestive symptoms including vomiting, diarrhea, nausea, and abdominal pain.

## Discussion

The contributions of our paper are multi-faceted. First, we performed a systematic survey on the phenotypes/symptoms on COVID-19 patients who got infected at the early stage of the pandemic (before early May 2020), from the publications reported before August 2020. Our study focused on the influence of three variables on the symptom outcomes: location of incidence, time of incidence, and comorbidity type. The ontology analysis allowed us to focus on groups of symptoms or comorbidities. Second, differential COVID-19 symptom patterns given different conditions were identified and analyzed. Our survey identified and classified 18 common symptoms. Furthermore, we found that these symptoms (e.g., loss of smell and taste) often differed significantly given specific regions, time, and comorbidities. Third, we showed how Coronavirus Infectious Disease Ontology (CIDO) can be applied to support modeling and linkage between COVID-19, viruses, symptoms, and comorbidities. Fourth, we propose a “Spiral Symptom Occurrence Hypothesis” to explain the spiral occurrence of certain symptoms that have occurred during the COVID-19 pandemic, as further discussed below. The first and third contributions listed here focus on method novelty, and the second and fourth contributions are scientifically more important.

It is generally considered that different SARS-CoV-19 variants induced similar symptoms, as demonstrated by the 18 common symptoms classified under different categories (Figure 1). However, symptoms might significantly differ given conditions. Many infected patients had no symptoms, but many died with severe symptoms, indicating human conditions significantly affected the disease outcome. On the other hand, virus variations might significantly change the disease outcome. Some variants, including Alpha variant (12) and Delta variant (59), cause more severe illness and death than the other variants. However, the more recent Omicron variant appears to cause milder symptoms in general (60, 61). Since our study focused on the early pandemic stage, we did not need to consider the influence of the highly transmissible virus variants reported at the later stage. To the best of our knowledge, our study provided the first systematic analysis of the influence and co-influence of three specific variations (i.e., location, time, and comorbidity type) on various COVID-19 symptoms. While the time variable is mainly reflected in the time when the data were collected, we focused on the variables of the location and comorbidity type.

The location of incidence at the early stage of pandemic appeared to significantly influence the disease symptoms. Many distinct and differential COVID-19 symptoms among different regions before the summer of 2020 were identified using our ontology-based literature meta-analysis (Table 1; Figures 2–4). From our comparison of individual symptoms in China, Italy, and the US, we found that COVID-19 patients in China frequently exhibited fever, whereas the USA ranked high in symptoms of cough, dyspnea, and sore throat, etc. And Italy was ranked high in myalgia/arthralgia, loss of smell, loss of taste, and fatigue (Figure 2). Overall, the patients in Italy and the USA had more similar symptoms than those in China. A possible reason for the difference could be different reporting styles of the disease between China and Italy/USA. However, it was likely not the case due to the fairly similar nature of professional investigations across different regions. The other possible reasons include viral variation, time of occurrence, host variation, non-pharmaceutical intervention policy, etc. (see more discussion below).

Grouped symptoms in the nervous system (including loss of smell, loss of taste, and headache) (Figure 3) and the abdominal symptom (including nausea, vomiting, abdominal pain, and diarrhea) (Figure 4) with more countries included were then further analyzed with the help of the HPO ontology classification. Our study found that more of the COVID-19 patients from Europe and USA had nervous symptoms and abdominal symptoms compared with patients from Asia (Figures 3, 4). For example, during the early pandemic stage, 51–52% of patients in Italy and 32–41% of patients in US reported the losses of smell or taste, and the numbers came below 6% in China. Diarrhea occurred in East Asian countries was lower than 20%. In contrast, the incidence in European countries and the USA generally ranged from 20 to 61% (Figure 4; Supplementary Files 1, 2).

Comorbidities have been strongly associated with COVID-19 patients' clinical outcomes (Figures 1B, 5). A total of 22 comorbidities (Figure 1B) were found to commonly exist among COVID-19 patients, and usually COVID-19 patients with comorbidities had worse outcomes. Patients with these comorbidities such as diabetes and kidney failure were found to have worse outcomes compared with those without these comorbidities (Figure 5). Specifically, the percentage of severe COVID-19 patients with diabetes was generally higher than that in non-severe patients (Figure 5). Our collected data also showed that in most regions, the percentage of kidney disease of COVID-19 patients was higher in severe patients than in non-severe patients (Figure 5). The incidence of acute kidney injury (AKI) in hospitalized patients varies from 0.5 to 40% due to factors such as race, region, and disease severity, and the mortality rate is significantly higher than the general infected population (1, 2, 54, 62–65). A recent study identified two clinical phenotypes of AKI (AKI-early and AKI-late) based on whether the kidney is the first dysfunctional organ (66). Such a distinction is likely due to whether the kidney is directly infected or indirectly affected through the failure of other infected organs. How the kidney disease as a comorbidity affects the host-coronavirus interaction and disease outcomes appears very complex and requires further investigation.

Many reasons may explain the symptom differences identified in the early stage of COVID-19 pandemic. First, data biases in the literature report may exist. For example, cases of some specific symptoms might have been reported more in some regions than the others. We knowledge that such biases might have existed. However, given the impact and scope of the pandemic, there had been a lot of high impact and professional case reports, and we managed to collect all we could have found. Therefore, the data biases were likely low. Second, different predominant viral strains and viral variation in different regions may be combined to affect the outcomes. As per Forster et al. (March 30, 2020), novel coronavirus strains can be divided into three types: A, B and C (9). Although the original version of “A type” appeared in Wuhan, there were more mutated “B type” strains in Wuhan samples. B type is more common in East Asia. Type C, a variant of type B, is mainly present in Europe and has been found in early French, Italian, Swedish and British patients. The type A strain was more common in the US and Australian study samples (9). However, there has been skepticism about the claim reported in the paper (9), considering that the sample size they collected was too small, only 160 cases. An examination of strains collected from Northern California in early February and mid-March 2020 indicated that international travel (from China and Europe) and interstate travel caused multiple transmissions. Sequencing of strains collected in the New York metropolitan area in March 2020 also suggested that the viral strains found in New York originated from Europe and other parts of the US (67). The results may explain the similarities of COVID-19 symptoms among European countries and the USA.

Our early-pandemic phenotypic analysis does not cover the potential influences of major genetic variants of concern or interest of COVID-19 after the first stage of pandemic. The WHO defined many variants of concern (VOCs) and variants of interest (VOIs) of SARS-CoV-2 worldwide (68). The comparison of our collected findings with the new reports of symptoms associated with the infection of the VOCs and VOIs suggests that the symptoms associated with these VOCs and VOIs are generally similar but may differ in specific scenarios. There is evidence that variants with the D614G mutation spread more quickly than viruses without this mutation (69). Variants containing the D614G mutation are found in the B.1 clade by the PANGOLIN tool. The Alpha variant (lineage B.1.1.7), first detected in September 2020 in United Kingdom, shows ~50% increased transmission (11). The Beta strain (lineage B.1.351) was first identified at South Africa in October 2020 and likely first emerged in July or August 2020 (13). The Delta variant (lineage B.1.617), first discovered in October 2020 in India, strain was dominant in many countries (70) for a long period of time in 2021. The most recent Omicron variant (lineage B.1.1.529) is more transmissible but likely less virulent (60, 61). The last day of the data collection for the papers included in our study was May 2, and those data came from Italy (31). Therefore, our study does not describe the specific impact of these VOCs and VOIs. Such a limitation can be filled up with more specific studies. However, our study design also offers us an advantage in that we could focus on the impact of other factors.

Our study did not specifically analyze the influence of many factors, such as host genetic variations, age, gender, vaccination, and non-pharmaceutical interventions, on the symptom outcomes in COVID-19 patients. More specific investigations are required to overcome these limitations. The host genetic variations may be reflected in the factor of different regions or countries. The FDA issues the emergency use authorization for the first COVID-19 vaccine, i.e., Pfizer-BioNTech COVID-19 Vaccine, in December 2020. Considering our study used the clinical data during the early stage of the pandemic, the impact of vaccination was not shown in our study. Different from vaccination and drug treatment, many non-pharmaceutical interventions (NPIs), such as social distancing, travel restriction, and resource allocation, are very effective against COVID-19 pandemic (71). During the pandemic, China took very strict NPI measures, but the levels of NPIs in other countries varied in time and location. The specific influence of the NPIs on the symptoms of COVID-19 was not analyzed in this study.

Instead of providing direct guidance on the COVID-19 disease management in the post-vaccination period, our study provides a more historical perspective of the disease symptoms and evolutions at the early stage of the COVID-19 outbreak and then pandemic. Such results can also be used to compare with the symptom data obtained during the later stages of the pandemic, leading to more scientific insights and hypotheses. As a demonstration, after putting together our early stage findings and the symptoms observed with the COVID-19 variants coming out at later stages, we have identified and are proposing here a new hypothesis to address the dynamic symptom changes given different conditions:

Our *Spiral Symptom Occurrence Model Hypothesis* proposes that while non-specific symptoms (e.g., fever and fatigue) are conserved and transmission rate is increasing, specific COVID-19 symptoms (e.g., loss of smell and taste) may spirally be absent, appear, and then disappear in patients, primarily through viral genetic variations and host responses under various conditions.

Aligned with this hypothesis, initially the loss of smell and loss of taste did not occur frequently in the very early stage of infection in China, then occurred a lot in the European and Mideast countries and U.S., and now they have apparently disappeared or at a much less frequency in patients infected with the Omicron variant (60, 61). More specifically, these two symptoms were below 6% in early 2020 in China, then they were significantly changed to the range of 32–75% at later time in the first half of 2020 in US and Europe (Table 1; Figure 4), and now both symptoms have not generally been reported from the COVID-19 patients infected with the Omicron variant (60, 61). Our proposed spiral model suggests that the viruses might have first randomly mutated into more virulent and transmissible variants leading to the appearance of the loss of smell and taste; however, given various human responses including vaccination and non-pharmaceutical interventions, the viruses further mutated to form genetic variants such as Omicron that failed to generate the same symptoms. Based on this hypothesis, it is possible to identify some specific genetic mutations responsible for the loss of smell and tastes, which may be present in strains like Alpha and Delta variants but be absent or disappear in the Wuhan and Omicron genomes.

In the future, we plan to perform more systematic monitoring and analysis of the symptoms and comorbidities in COVID-19 patients of dominant variants (e.g., Alpha, Delta, and Omicron) given various conditions. We also plan to further compare our results found at the early stage of the pandemic with the later stages of the pandemic. Our integrative ontology-based analysis approaches as demonstrated in this paper provide a new strategy for data standardization, normalization, and systematic comparison. Our two newly generated relations (Figure 6) allowed semantic and accurate presentation of the relations between COVID-19 and its associated 18 symptoms and 22 comorbidities. Such representations then allow the development of new computer queries and software programs, which further advance our opportunities for more advanced analyses. To better understand the contributions of different factors, we will also require deep modeling and representation of intricate molecular and cellular host-coronavirus interactions and understanding the fundamental interaction mechanisms.

## Conclusions

The clinical data of COVID-19 phenotypes found in 43,567 COVID-19 patients, as reported in 48 articles, were systematically surveyed and analyzed. The 18 commonly occurring symptoms and 22 comorbidities in COVID-19 patients were ontologically categorized and grouped using the Human Phenotype Ontology (HPO). Differential patterns were detected in different countries and regions. Fever, cough, and the new loss of smell/taste were ranked as the most frequent phenotypes in China, the US, and Italy, respectively. Using data reported from 12 countries, we found that the patients from Europe and USA more frequently had nervous system phenotypes and abdominal phenotypes than patients from Asia during the first half of 2020. The Coronavirus Infectious Disease Ontology (CIDO) was used to model and interlink the COVID-19 disease, 18 symptoms, and 22 comorbidities together, and CIDO-based semantic queries were performed to demonstrate the advantages of computer-assisted reasoning and analysis. In addition to our method novelty, these findings discovered in this study provide historic views of COVID-19 symptoms in terms of our long-term COVID-19 disease study.

## Data Availability Statement

The original contributions presented in the study are included in the article/Supplementary Material, further inquiries can be directed to the corresponding author/s.

## Author Contributions

YW, XY, and HY contributed to the overall study design. YW, FZ, and HY contributed to data collection and data analysis. YW and YH provided manual data/result verification and ontology editing. JB, HY, and XY who are COVID-19 medical doctors, served as domain experts to perform data/result interpretation, and discussion. YW and YH prepared the initial complete manuscript and all authors contributed to the manuscript writing and reviews. All authors contributed to the article and approved the submitted version.

## Funding

YW was supported by a scholarship offered by the China Scholarship Council with the University of Michigan as a Visiting Ph.D. Student (File No. 202006670006). The research was also supported by the Youth Found of Guizhou Provincial People's Hospital of China, GZSYQN[2019]09, a non-profit Central Research Institute Fund of the Chinese Academy of Medical Sciences (2019PT320003), a NIH NIAID grants 1UH2AI132931 (to YH), a COVID-19 pilot award from Michigan Medicine–Peking University Health Sciences Center Joint Institute for Clinical and Translational Research (to YH), and a Fast Grants award for a clinical trial related to COVID-19 (to JB).

## Conflict of Interest

The authors declare that the research was conducted in the absence of any commercial or financial relationships that could be construed as a potential conflict of interest.

## Publisher's Note

All claims expressed in this article are solely those of the authors and do not necessarily represent those of their affiliated organizations, or those of the publisher, the editors and the reviewers. Any product that may be evaluated in this article, or claim that may be made by its manufacturer, is not guaranteed or endorsed by the publisher.
