# Supplementary material for: Differential COVID-19 Symptoms Given Pandemic Locations, Time, and Comorbidities During the Early Pandemic
Source: Front Med (Lausanne). 2022 Jan 28;9:770031. doi: 10.3389/fmed.2022.770031 (PMC8831795; doi:10.3389/fmed.2022.770031)
Supplement: Supplementary file 4 [file Table_4.docx]

**Supplemental Table 1.** **Common symptoms/phenotypes and comorbidities of COVID-19 based on Human Phenotype Ontology (HPO) classification.**

|  | **COVID-19 Symptoms / Phenotypes or Comorbidities (HPO term IDs)** | | | |
| --- | --- | --- | --- | --- |
| **18 symptoms/ phenotypes in COVID-19 patients** | Fever  (HP_0001945)  Cough  (HP_0012735)  Nasal congestion (HP_0001742)  Coagulation disorder(HP_0001928) | Dyspnea (HP_0002094)  Headache  (HP_0002315)  Abdominal pain (HP_0002027)  Sore throat (HP_0100776) | Myalgia (HP_0003326)  Chills (HP_0025143)  Fatigue  (HP_0012378)  New loss of smell or taste (HP_0000458,HP_0031249) | Arthralgia (HP_0002829)  Diarrhea  (HP_0002014)  Rhinorrhea  (HP_0031417)  Nausea and vomiting  (HP_0002018, HP_0002013) |
| **22 common**  **comorbidities in COVID-19 patients** | Immunodeficiency  (HP_0004430)  Congestive heart failure  (HP_0001635)  Abnormality of blood and blood-forming tissues  (HP_0001871)  Hypercholestero-lemia  (HP_0003124)  Obstructive sleep apnea  (HP_0002870) | Hepatitis  (HP_0006562)  Allergic rhinitis  (HP_0003193)  Asthma  (HP_0002099)  Diabetes mellitus  (HP_0000819)  Obesity  (HP_0001513)  Neoplasm  (HP_0002664)  Status post organ transplantation  (HP_0032444) | Cirrhosis  (HP_0001394)  Chronic hepatic failure  (HP_0100626)  Malnutrition  (HP_0004395)  Dementia  (HP_0000726)  Seizure  (HP_0001250)  Rheumatoid arthritis  (HP_0001370) | Hypertension  (HP_0000822)  Chronic kidney disease  (HP_0012622)  Chronic obstructive pulmonary disease  (HP_0006510)  Chronic neurological disease  (HP_0000707) |

***Note***: This information has been used in Figure 1 (A) and (B).
